# Quantification and Validation of Measurement Uncertainty in the ISO 8192:2007 Toxicity Assessment Method: A Comparative Analysis of GUM and Monte Carlo Simulation

**DOI:** 10.3390/toxics13100857

**Published:** 2025-10-10

**Authors:** Bettina Neunteufel, Dirk Muschalla

**Affiliations:** Institute of Urban Water Management and Landscape Water Engineering, Graz University of Technology, Stremayrgasse 10/I, 8010 Graz, Austria; d.muschalla@tugraz.at

**Keywords:** measurement uncertainty, GUM method, Monte Carlo Simulation, ISO 8192:2007, uncertainty analysis

## Abstract

Reliable toxicity assessments are essential for protecting biological processes in wastewater treatment plants (WWTPs). This study focuses on quantifying the measurement uncertainty of the ISO 8192:2007 method, which determines the inhibition of oxygen consumption in activated sludge. Using the GUM guideline and Monte Carlo Simulation (MCS), up to 29 uncertainty contributions were evaluated in terms of oxygen consumption rate and percentage inhibition. The results reveal that temperature tolerance, measurement interval, and oxygen probe accuracy are dominant contributors, accounting for over 90% of the total uncertainty. The GUM results for oxygen consumption rates were validated by Monte Carlo Simulation, confirming their reliability. The percentage inhibitions showed asymmetric distributions and were underestimated by the GUM method, especially at lower toxicant concentrations. This highlights the necessity of simulation-based approaches for asymmetric systems. Notably, the consideration of correlations in the GUM analysis had minimal impact on outcomes. The findings emphasize the need for the precise recording of measurement time intervals, temperature control, the regular calibration of oxygen probes, and repeat measurements at low toxicant concentrations. Overall, this study enhances the robustness of ISO 8192:2007-based toxicity testing and provides practical guidance for reducing measurement uncertainty.

## 1. Introduction

The determination of substance toxicity using the ISO 8192:2007 method (a method for determining oxygen consumption inhibition in activated sludge) [1] plays a crucial role in environmental analytics. This method is used to assess potentially toxic effects on microbiological processes and is, therefore, an essential tool for protecting the biological processes in wastewater treatment. The method determines the EC_50_ value for the percentage of inhibition of total oxygen consumption, heterotrophic oxygen consumption inhibition, and the percentage inhibition of oxygen consumption by nitrification.

However, the method is subject to significant variations and measurement uncertainties, which can substantially affect the measurement results. The variability in biological processes, measurement device tolerances, and environmental factors such as temperature contribute to these uncertainties. In a previously conducted study by Neunteufel et al. [2], the sensitive boundary conditions for toxicity determination were identified. The present study quantifies measurement uncertainties to improve the reliability and comparability of the results, thereby contributing to a more accurate assessment of ecotoxicological risks in wastewater treatment plants.

The ISO 8192:2007 method [1] encompasses a broad validity range for the different inhibitions. The total oxygen consumption range of validity is 2–25 mg/L, the heterotrophic oxygen consumption range of validity is 5–40 mg/L, and the nitrification inhibition range of validity is 0.1–10 mg/L.

These wide ranges likely reflect the natural variability in biological processes and differences in wastewater compositions. Consequently, quantifying uncertainty is essential to distinguishing real toxic effects from measurement variability. This is especially important in sensitive areas, where an underestimation of measurement uncertainty could result in hazardous substances being misclassified as harmless. In such cases, measured values may fall below legal threshold limits, not because the substances are truly safe, but due to unrecognized uncertainty. Such misclassifications are particularly problematic for wastewater treatment plants because their performance depends on the activity and stability of microorganisms that cope with fluctuations in wastewater composition [3]. A sudden increase in certain compounds can reduce microbial activity [4] and, in severe cases, lead to system failure [3,5,6,7] and harm the aquatic environment. This underscores the necessity of a measurement uncertainty analysis to enhance the reliability and comparability of toxicity assessments. A realistic estimate of uncertainty is crucial to avoid wrong decisions in environmental and water protection.

Measurement uncertainty is a key indicator to express measurement results and reliability [8]. It allows for an objective assessment of different measurement results and ensures their comparability between different laboratories [8]. It is a general requirement of ISO/IEC 17025 [9] which is used for the accreditation of laboratories. In addition to laboratory practice, the evaluation and transparent reporting of measurement uncertainties are also important in a regulatory context. ISO/IEC 17025 [9] ensures that ecotoxicological test results are reliable and comparable between laboratories. Therefore, uncertainty analysis is crucial not only for the operation of wastewater treatment plants, but also for compliance with comprehensive environmental protection regulations.

All measurements are subject to a certain degree of uncertainty that can arise, for example, from the precision of the measuring instrument [10], the environment, and human repeatability [11]. Evaluating this uncertainty, also known as uncertainty analysis or error analysis, is essential for the credibility of scientific conclusions [10].

It is standard practice to differentiate between three categories of error: constant errors (e.g., faulty calibration of a measuring instrument), systematic errors (e.g., zero errors on screw gauge), and random errors (e.g., fluctuations in environmental conditions) [10]. Cotman and Pintar [12] demonstrated the impact of errors: in their study, sampling uncertainty alone accounted for 29% of the total measurement uncertainty.

The “Guide to the Expression of Uncertainty in Measurement” (GUM) [13] is an internationally recognized approach to the estimation of measurement uncertainties developed by ISO and has received worldwide distribution and acceptance [14,15,16,17]. El Fazani et al. [18], Haidara et al. [19], and Lee et al. [20] demonstrate the wide range of applications of this method. The GUM method can be used for a linear model with multiple input quantities and a single output quantity and is based on the law of uncertainty propagation and a characterization of the output quantity by a normal distribution or a t-distribution [21]. If the model is not linear or if the output variables deviate from the normal distribution or t-distribution due to marked asymmetries, Monte Carlo Simulation is a recommended alternative for validating GUM results [21]. Supplement 1 [21] was developed for this application and has been used in many studies [8,14,22,23,24,25,26].

In the literature [27,28,29], comparative values of ISO 8192:2007 [1] for 3,5-dichlorophenol can be found, but the associated measurement uncertainties are not discussed. This study therefore aims to close this gap. The objective was to analyze the measurement uncertainties of the method for determining oxygen consumption inhibition in activated sludge, based on ISO 8192:2007 [1], and the modified version described by Neunteufel et al. [2] using the GUM methodology [13] and to validate the results through Monte Carlo Simulation [21]. The following research questions will be addressed:

- What is the measurement uncertainty of the toxicity method when calculation is carried out using the GUM method (with and without correlations)?
- How well do the results of the GUM method align with those of Monte Carlo Simulation?

## 2. Methodology

### 2.1. Test Setup and Measurement Procedure for the Activated Sludge Respiration Inhibition Test

**Figure 1:**
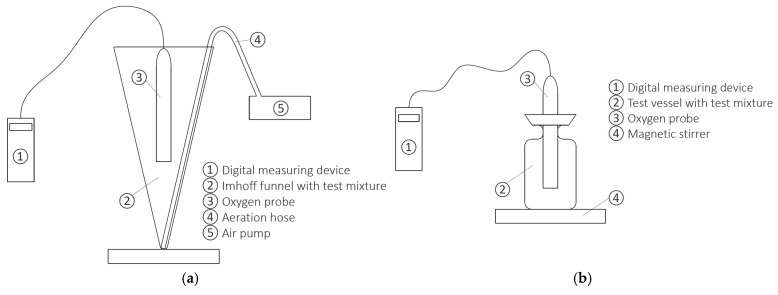
Test setup and measurement procedure for the activated sludge respiration inhibition test based on ISO 8192:2007 [1] and the modification reported by Neunteufel et al. [2]. (**a**) Equipment and experimental setup required for aeration; (**b**) devices and setup for measuring oxygen concentrations.

The experimental setup and measurement procedure were established according to ISO 8192:2007 [1] and the modification described by Neunteufel et al. [2]. For the investigations, nitrified activated sludge from the wastewater treatment plant of the city of Graz, Austria was utilized, with 3,5-dichlorophenol serving as the reference substance, as recommended in ISO 8192:2007 [1]. Even if other reference substances are specified in the ISO 8192:2007 [1], 3,5-dichlorophenol was chosen because it is also specified as a reference substance in other standards, for example, in [30,31,32,33,34], as compared in Strotmann et al.’s work [29]. This ensures the international comparability of different methods. The activated sludge was allowed to settle at room temperature for approximately one hour, before being decanted, and the supernatant was replaced with chlorine-free tap water. This process was repeated four times to clean the activated sludge. In order to determine the inhibitions, the following test medium needed to be prepared: 16 g of peptone (Karl Roth GmbH + Co. KG, Karlsruhe, Germany), 11 g of meat extract (Karl Roth GmbH + Co. KG, Karlsruhe, Germany), 3 g of urea (MERCK, Darmstadt, Germany), 0.7 g of sodium chloride, 0.4 g of calcium chloride dihydrate (MERCK, Darmstadt, Germany), 0.2 g of magnesium sulphate heptahydrate (MERCK, Darmstadt, Germany), 2.8 g of anhydrous potassium dihydrogen phosphate (MERCK, Darmstadt, Germany) and 1 L of distilled/deionized water. In addition, N-allylthiourea (ATU) (MERCK-Schuchardt, Hohenbrunn, Germany) needed to be dissolved at a concentration of 2.5 g per 1000 mL of distilled/deionized water, as did 3,5-dichlorophenol (SIGMA-ALDRICH Co., St. Louis, MO, USA) at a concentration of 1 g per 1000 mL of distilled/deionized water. According to ISO 8192:2007 [1] recommendations, a test mixture with different dilution levels was prepared (with at least three test material concentrations, e.g., 1.0 mg/L, 10 mg/L and 100 mg/L, and a blank control). Four additional dilution levels were prepared to enable more meaningful inhibition curves to be displayed. After aerating (600 L/h, SuperFish; and 300 L/h, JBL, Neuhofen, Germany) the test mixture for 30 min, it was transferred into a test vessel placed on a magnetic stirrer (Rotilabo MH 15 Karl Roth GmbH + Co. KG, Karlsruhe, Germany). Oxygen consumption was measured in the test vessel using an oxygen probe (FDO 925 WTW, Weilheim, Germany; and Multi 3430 WTW, Weilheim, Germany). The experimental setup required the test environment and the test mixture to be maintained at a temperature of 22 ± 2 °C, and the pH of the test medium kept to 7.5 ± 0.5. Evaluation of the experiments was performed by linear regression of the oxygen consumption curves (which occurs at an oxygen concentration between approximately 2 and 7 mg/L), following prior outlier identification and removal using Cook’s Distance. Finally, inhibition curves were generated to determine the EC_50_ value. Figure 1a,b provide an overview of the required equipment and the experimental setup for aeration, as well as the measurement setup for determining the oxygen concentration.

Calculation was performed in accordance with ISO 8192:2007 [1] and the modification reported by Neunteufel et al. [2]:

The oxygen consumption rate was calculated as follows:

(1) $$R_{i}=\left(\frac{\rho_{1}-\rho_{2}}{∆t}\right)*60\;\left[mg/Lh\right]$$

where $\rho_{1}$ represents the oxygen concentration at the beginning of the relevant range (mg/L), $\rho_{2}$ is the oxygen concentration at the end of the relevant range (mg/L), and ∆t is the time interval (min).

The percentage inhibition of total oxygen consumption was calculated as follows:

(2) $$I=\left[1-\frac{R_{T}}{R_{TB}}\right]*100\;[\%]$$

with $R_{T}$ as the oxygen consumption of the respective test mixture (mg/Lh) and $R_{TB}$ as the oxygen consumption of the blank control (mg/Lh).

The percentage inhibition of heterotrophic oxygen uptake was calculated as follows:

(3) $$I_{H}=\left[1-\frac{R_{H}}{R_{HB}}\right]*100\;[\%]$$

where $R_{H}$ represents the oxygen consumption of the respective heterotrophic test mixture (mg/Lh), and $R_{HB}$ is the oxygen consumption of the heterotrophic blank control (mg/Lh).

The percentage inhibition of oxygen uptake due to nitrification was calculated as follows:

(4) $$I_{N}=\left[1-\frac{R_{T}-R_{H}}{R_{TB}-R_{HB}}\right]*100\;[\%]$$

Finally, inhibition curves were generated to determine the EC_50_ value. To assess measurement uncertainty, measurement results from a total of 30 (distinct, non-consecutive days) measurement days from the years 2022 to 2024 were considered. In 2024, monthly measurements were carried out, each of which was supplemented by repeat measurements the following day. Additionally, reference measurements with 3,5-dichlorophenol were recorded as part of tests involving industrial wastewater. The higher number of blank samples is due to duplicate measurements being carried out in each case.

Table 1 shows the number of oxygen consumption rate measurements per concentration of 3,5-dichlorophenol.

### 2.2. Principles of the GUM Method

The determination of measurement uncertainty using the GUM method was carried out in accordance with Joint Committee for Guides in Metrology (JCGM) 100:2008 [13] and can be summarized as follows:

1. Documentation of all relevant input quantities (direct measurement results, manufacturer specifications, the literature values, …) to determine the model equation.
  (5) $$Y=f(X_{1},X_{2},\ldots ,X_{N}),$$
  The measurand Y is influenced by the input quantities $X_{i}$, which are incorporated into the model equation.
2. We determined the estimates $x_{i}$ and the corresponding standard uncertainties $u\left(x_{i}\right)$ for each input quantity. A distinction was made between two types of uncertainty. Type A: Statistical analysis of repeated measurements. Type B: Uncertainties derived from manufacturer specifications, calibration data, or literature values.
3. Definition of the probability density function. For Type A evaluation, a normal distribution is applied when the number of repeated measurements n>20 [35]. In this case, the standard uncertainty $u\left(x_{i}\right)$ is calculated from the standard deviation $s\left(x_{i}\right)$ of the mean value of n measurements. For Type B evaluations, in this study, only manufacturer specifications with fixed tolerances were considered. As only +/− values from the manufacturer’s specifications are known, a rectangular distribution was used to determine the standard uncertainty $u\left(x_{i}\right)$ according to JCGM 101:2008 [21].
4. Calculation of the uncertainty contributions $u_{i}\left(y\right)$ by multiplying the standard uncertainty $u\left(x_{i}\right)$ with the corresponding sensitivity coefficient $c_{i}$.
  (6) $$u_{i}\left(y\right)=u\left(x_{i}\right)c_{i}\mathrm{with}\;c_{i}=\frac{\partial f}{\partial Xi}$$
5. The combined standard uncertainty $u\left(y\right)$ is determined either without considering correlations by summing the squared uncertainty contributions $u_{i}\left(y\right)$,
  (7) $$u\left(y\right)=\sqrt{\sum_{i=1}^{n}{c_{i}}^{2}u^{2}\left(x_{i}\right)}$$
  or with correlations by additionally accounting for correlation terms between input quantities.
  (8) $$u\left(y\right)=\sqrt{\sum_{i=1}^{n}{c_{i}}^{2}u^{2}\left(x_{i}\right)+2\sum_{i=1}^{n-1}\sum_{j=i+1}^{n}c_{i}c_{j}u\left(x_{i}\right)u\left(x_{j}\right)r(x_{i},x_{j})}\;\mathrm{with}\;r(x_{i},x_{j})=\frac{u\left(x_{i},x_{j}\right)}{u\left(x_{i}\right)u\left(x_{j}\right)}\;\mathrm{and}\;u\left(x_{i},x_{j}\right)=\frac{1}{n\left(n-1\right)}\sum_{k=1}^{n}\left(x_{i,k}-\bar{x_{i}}\right)(x_{j,k}-\bar{x_{j}})$$
  In Equation (8), $r(x_{i},x_{j})$ represents the correlation coefficient, and $u\left(x_{i},x_{j}\right)$ is the covariance between $x_{i}$ and $x_{j}$, calculated from the individual measurement values $x_{i,k}$ and $x_{j,k}$. The terms $x_{i}$ and $x_{j}$ denote the arithmetic means of the respective measurement series.
6. Calculation of the expanded measurement uncertainty U(y) using an expansion factor of k=2, corresponding to a coverage interval of 95%.
  (9) U(y)=k∗u(y)

### 2.3. Principles of the Monte Carlo Simulation

The Monte Carlo Simulation was conducted in accordance with JCGM 101:2008 [21] and can be summarized as follows:

1. Documentation of all relevant input quantities (direct measurement results, manufacturer specifications, the literature values, …) to determine the model equation. The model equation corresponds to that of the GUM method.
2. Determination of the probability density functions of the input quantities. These functions were adopted from the GUM method. JCGM 101:2008 [21] represents an approach that is as consistent as possible with the GUM method, especially through the uniform use of probability density functions. For example, also the study by Chen and Chen [8] was conducted in accordance with the specified protocol.
3. Creation of the covariance matrix $U_{x}$ of dimension N×N to take account of correlations between input quantities:
  (10) $$U_{x}=\left[\begin{array}{ccc} u\left(x_{1},x_{1}\right) & \ldots & u\left(x_{1},x_{N}\right)\\ \vdots & \ddots & \vdots \\ u\left(x_{N},x_{1}\right) & \ldots & u\left(x_{N},x_{N}\right) \end{array}\right]$$
  where $u\left(x_{i},x_{i}\right)=u^{2}\left(x_{i}\right)$ is the variance and $u\left(x_{i},x_{j}\right)$ the covariance.
4. Selection of the number M of Monte Carlo trials. The number was set to $M=10^{6}$. According to JCGM 101:2008 [21], at $M=10^{6}$ the 95% coverage interval is typically accurate to one or two significant decimal digits.
5. Calculation of the M simulation results.
6. Sorting of the calculated values in non-decreasing order to determine the interval limits $y_{low}$ and $y_{high}$. This allows for a direct comparison of the computed 95% coverage interval with the k=2 standard uncertainty from the GUM analysis.

### 2.4. Validation of the GUM Using the Monte Carlo Simulation

1. Calculation of the absolute differences in the respective endpoints of the coverage intervals of GUM method and Monte Carlo Simulation:
  (11) $$d_{low}=\left|\left(Y-U(y)\right)-y_{low}\right|$$
  (12) $$d_{high}=\left|\left(Y+U(y)\right)-y_{high}\right|$$
2. Calculation of δ to JCMG 101:2008, where δ is the numerical tolerance:
  (13) $$\delta =\frac{1}{2}10^{l}$$
  δ is calculated by expressing the combined standard uncertainty U(y) obtained by the GUM in the form $c\;\times \;10^{l}$, where c has the same number of significant decimal digits regarded as meaningful in $U\left(y\right)$, and l is an integer. For example, $U\left(y\right)\;=\;0.8\;mg/Lh$, and $U\left(y\right)$ can be expressed as $8\;\times \;10^{-1}\;mg/Lh$, and so c=8 and $l=10^{-1}$. Take $\delta =\frac{1}{2}10^{-1}=0.05\;mg/Lh$.
3. If $d_{low}<\delta$ and $d_{high}<\delta$, the comparison is favorable and GUM has been validated in this instance.

## 3. Mathematical Modeling and Identification of Uncertainties

### 3.1. Error Analysis

**Figure 2:**
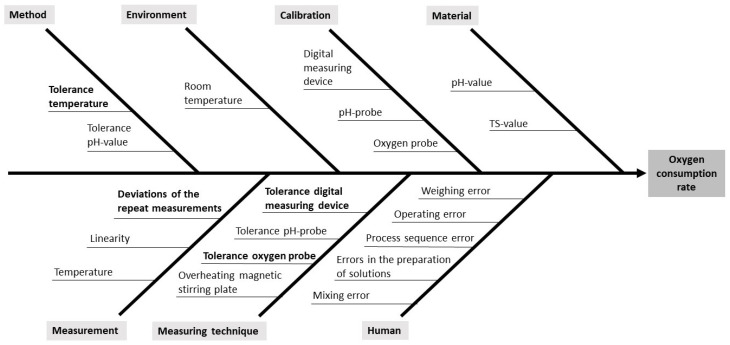
Ishikawa diagram showing the various influences on the result of the oxygen consumption rate. The influences printed in bold are taken into account for the model equation.

To identify all relevant uncertainties and establish the model equation, a cause-and-effect diagram (Ishikawa diagram) was created for error analysis; see Figure 2.

In a further step, the remaining uncertainties were analyzed based on the results of the error analysis. Relevant tolerance ranges according to the ISO 8192:2007 method [1], manufacturer specifications of the measuring instruments, and deviations from repeated measurements were considered. All other uncertainties could be eliminated before measurement and therefore did not need to be included in the uncertainty calculation. This is achieved through rigorous checks and controls of the test environment, instruments, and trained personnel. All measurements were carried out using the same calibrated device, and the test setup (aeration, mixing, and the measurement protocol) remained unchanged throughout the study. The following parameters are included in the model equation:

**Method ISO 8192:2007**

1. Tolerance temperature $\delta_{TT}=\pm 2\;{}^{\circ}C$.

**Tolerance digital measuring device (Multi 3430 WTW, Weilheim, Germany)**

2. Accuracy of the oxygen concentration: $\delta_{\rho 1,S},\delta_{\rho 2,S}=\pm 0.03\;mg/L$
3. Accuracy of the temperature: $\delta_{TS}=\pm 0.5\;{}^{\circ}C$

**Tolerance oxygen probe (FDO FDO 925 WTW, Weilheim, Germany)**

4. Accuracy of oxygen measurement at 20 °C: $\delta_{\rho 1,G},\delta_{\rho 2,G}=\pm 1.5\%$
5. Accuracy of the temperature measurement: $\delta_{TG}=\pm 0.2\;{}^{\circ}C$

**Deviations of the repeat measurements**

6. $\rho_{1}$: oxygen concentration at the beginning of the relevant range (mg/L).
7. $\rho_{2}$: oxygen concentration at the end of the relevant range (mg/L).
8. ${∆}_{t}$: time interval (min).
9. ${∆}_{T}$: Temperature (°C).

Table 2 assigns these parameters to the respective uncertainty type according to the GUM method, describes their distribution, and presents the calculation of the standard uncertainty according to GUM.

### 3.2. Correlations

For $\delta_{\rho 1,G}$ and $\delta_{\rho 2,G}$, as wells as for $\delta_{\rho 1,S}$ and $\delta_{\rho 2,S}$, a correlation coefficient of r=1 was estimated, because these input quantities were obtained using the same measurement device and are associated with common tolerance specification. The correlation coefficient for $\rho_{1}$ and $\rho_{2}$ was determined based on the measured data (Equation (8)), which was measured using the same measurement device. This is a realistic estimation of correlation coefficient in accordance with JCGM 100:2008 [13].

### 3.3. Model Equation

To account for temperature uncertainties, Equation (1) was extended by an additional temperature term:

(14) $$R_{i}=\left(\frac{{(\rho }_{1}-\rho_{2})*(1+\alpha *{∆}_{T})}{{∆}_{t}}\right)*60\;\left[mg/Lh\right]$$

The temperature correction coefficient, Θ=1.024, leading to $\alpha =0.024\;{{}^{\circ}C}^{-1}$, was chosen because it has been described several times in the literature as a typical value for biological wastewater processes. Myszograj (2018) [36] states that the value for the thermal decomposition of activated sludge is 1.024, based on a reference temperature of 20 °C, which corresponds to our test temperature. Additionally, the same value (Θ=1.024) is given in water quality modelling [37] as a typical oxygen input coefficient. Therefore, we used a value from the literature that is representative of both activated sludge under field conditions and established models. Considering the uncertainties, the following model equation is obtained:

(15) $$R_{i}=\left(\frac{{((\rho }_{1}+\delta_{\rho 1,G}+\delta_{\rho 1,S})-{(\rho }_{2}+\delta_{\rho 2,G}+\delta_{\rho 2,S}))*(1+\alpha *{(∆}_{T}+\delta_{TT}+\delta_{TS}+\delta_{TG}))}{{∆}_{t}}\right)*60\;\;\left[mg/Lh\right]$$

## 4. Results and Discussions

The present study was carried out using activated sludge from a single wastewater treatment plant (WWTP). This was a deliberate decision, as mixing sludge from different sources would obscure site-specific microbial characteristics and introduce variability that could not be distinguished from measurement uncertainty. Consequently, the results presented here are specific to the microbial community composition and operational conditions of the plant in question. However, the approach outlined in this study can be used as a framework to evaluate uncertainties in other WWTP systems. A systematic comparison of uncertainty estimates across different sludge sources would be a valuable area for future research.

Since the sample sizes were obtained through monthly measurements, supplementary repetitions the following day, and additional reference tests, a formal power analysis was not performed. However, there were more than 20 repetitions available for all concentration levels, justifying the application of a normal distribution according to GUM. The measurements also fall within the scope of ISO 8192:2007 [1], which underscores the methodological robustness. The risk of limited validity is low because the number of repetitions exceeds the minimum requirement, critical concentrations were tested frequently, and the results were validated using a Monte Carlo Simulation. Nevertheless, a power analysis would clarify type I and type II errors, so it should be carried out in future work to strengthen the results’ significance.

The biological properties of activated sludge fluctuate naturally, meaning no two days are alike. For this reason, measurements were taken over a long period of time and across all seasons to capture the full range of variability. Although limiting the measurements to a short period during which the sludge characteristics would have remained constant would have produced more homogeneous data, it would have greatly limited the information content and prevented the generalization of results for practical applications. Since no separate trend analysis was performed to investigate systematic, time-dependent effects, longer-term changes in sludge activity cannot be ruled out.

The temperature correction coefficient, θ = 1.024, was chosen based on typical values found in the literature for biological wastewater processes. It corresponds to standard specifications for the thermal degradation of activated sludge at 20 °C. For even more accurate results, however, it may be useful to check this value for the specific location and adjust it if necessary.

### 4.1. Analysis of Uncertainty Using the GUM Method

The following sections present the uncertainty budget for the oxygen consumption rate and the percentage inhibition, both with and without consideration of correlations between the input quantities. Due to the minor deviation and fluctuations observed between the uncorrelated and correlated results, the percentage contributions to the uncertainties were calculated only for the uncorrelated case.

#### 4.1.1. Oxygen Consumption Rate

In the assessment of the measurement uncertainty for the oxygen consumption rate (total respiration rate and rate due to heterotrophic respiration), based on ISO 8192:2007 [1] and Neunteufel et al.’s modification [2], as well as the application of the GUM methodology [13], a total of 11 sources of uncertainty (input parameters) were considered. Table 3 presents the uncertainty budget of the oxygen consumption rate (total respiration rate) using an example concentration of 3,5-dichlorophenol at 10 mg/L. On average, the three main sources of uncertainty contribute approximately 92% of the total uncertainty across all concentration levels.

Table 4 shows the uncertainty budget for the oxygen consumption rate (rate due to heterotrophic respiration) for a 3,5-dichlorophenol concentration of 10 mg/L. Here, the three main uncertainty sources account for 94% of the total uncertainty on average across all concentration levels.

The results indicate that three primary factors account for the majority of overall uncertainty, approximately 92% for total respiration and 94% for heterotrophic respiration. The primary sources of uncertainty are the same for both respiration rates. The greatest influence on overall uncertainty comes from the time interval ${∆}_{t}$ of the measurements, which significantly affects overall uncertainty. It contributes 34.8% to the uncertainty in total respiration rate and 49% to the uncertainty in heterotrophic respiration rate. This highlights that such high standard deviations lead to considerable variations in the final results, which can be attributed to differences in the composition of biological sludge. To minimize process variability, sludge pre-treatment was consistently carried out in accordance with ISO 8192:2007 [1] (sedimentation, decanting, and refilling with chlorine-free tap water). All measurements were carried out using the same calibrated device, and the test setup (aeration, mixing, and the measurement protocol) remained unchanged throughout the study. Despite these measures, it cannot be ruled out that operational changes at the sewage treatment plant or other external influences may have affected the sludge properties over the two-year period. As these factors were not systematically recorded, we primarily attribute the observed variance in ${∆}_{t}$ to biological variability. Despite this limitation, the results can still be considered a realistic representation of the uncertainties.

Another key source of uncertainty is the tolerance temperature $\delta_{TT}$, as defined in ISO 8192:2007 [1]. It accounts for 47.9% of the uncertainty in total respiration rate and 37.5% in heterotrophic respiration rate. This emphasizes the high sensitivity of the oxygen consumption rate to temperature fluctuations and underscores the necessity of strict temperature control during experiments. Since temperature variations can influence microbial respiration rates, maintaining stable conditions is crucial for obtaining reliable results. The third significant uncertainty factor is the accuracy of oxygen concentration measurements $\delta_{\rho 1,G}$ at the beginning of the relevant measurement range. This uncertainty is influenced by the precision of the oxygen probe, with uncertainty contributions ranging between 7.7% and 9.9%, depending on the parameter. The findings suggest that careful calibration and regular maintenance of oxygen probes are essential to minimize uncertainties in respiration measurements.

#### 4.1.2. Percentage Inhibition

In the assessment of the measurement uncertainty of the percentage inhibition of total oxygen consumption and heterotrophic oxygen uptake, based on ISO 8192:2007 [1] and Neunteufel et al.’s modification [2], as well as the application of the GUM methodology [13], a total of 17 sources of uncertainty (input parameters) were considered. Table 5 presents the four main uncertainties of the percentage inhibition of total oxygen consumption using an example concentration of 3,5-dichlorophenol at 10 mg/L. The source “Others” consists of 13 sources of uncertainties. On average, the main sources of uncertainty contribute approximately 94% of the total uncertainty across all concentration levels.

Table 6 shows the uncertainty budget for the percentage inhibition of heterotrophic oxygen uptake for a 3,5-dichlorophenol concentration of 10 mg/L. The source “Others” consists of 13 sources of uncertainties. Here, the four main uncertainty sources account for 96% of the total uncertainty on average across all concentration levels.

The primary sources of uncertainty are the same for both percentage inhibition of total oxygen consumption, approximately 94%, and heterotrophic oxygen uptake, approximately 96%, reflecting the outcomes observed for the oxygen consumption rate. The largest influence on overall uncertainty comes from the time interval ${∆}_{t,H}$ of the measurements at a concentration of 3,5-dichlorophenol. The second large influence comes from the time interval ${∆}_{t,\;FB,H}$ of the blank control measurements. This is followed by the accuracy of the oxygen concentration measurement at the beginning of the relevant range $\delta_{\rho 1,G}$ (blank control measurement and measurements with a concentration of 3,5-dichlorophenol), determined by the accuracy of the oxygen probe.

In the assessment of the measurement uncertainty of the percentage inhibition of oxygen uptake due to nitrification, based on ISO 8192:2007 [1] and Neunteufel et al.’s modification [2], as well as the application of the GUM methodology [13], a total of 29 sources of uncertainty (input parameters) were considered. The main uncertainties differ depending on the concentration. For inhibition with a low concentration of 3,5-dichlorophenol, there are seven main uncertainties, and for inhibition with a high concentration of 3,5-dichlorophenol, there are four main uncertainties.

Table 7 presents the four main uncertainties of the percentage inhibition of total oxygen consumption using an example concentration of 3,5-dichlorophenol at 10 mg/L. The source “Others” consists of 25 sources of uncertainties. On average, the main sources of uncertainty contribute approximately 96% (concentration of 3,5-dichlorophenol at 10–100 mg/L) of the total uncertainty.

Table 8 shows the uncertainty budget for the percentage inhibition of oxygen uptake due to nitrification for a 3,5-dichlorophenol concentration of 0.1 mg/L. The source “Others” consists of 22 sources of uncertainties. Here, the seven main uncertainty sources account for 93% (concentration of 3,5-dichlorophenol at 0.1–2 mg/L) of the total uncertainty.

Also, for this inhibition, the main uncertainties are the time interval of the measurements and the accuracy of the oxygen concentration measurement at the beginning of the relevant range, which is determined by the accuracy of the oxygen probe. However, it is evident that the uncertainties inherent in the blank samples exert a substantial influence on measurements conducted with low concentrations of 3,5-dichlorophenol. This can be attributed to the standard deviation of the observed measurements, which were found to be considerably higher at higher concentrations.

### 4.2. Validation of the GUM Using the Monte Carlo Simulation

#### 4.2.1. Oxygen Consumption Rate

**Figure 3:**
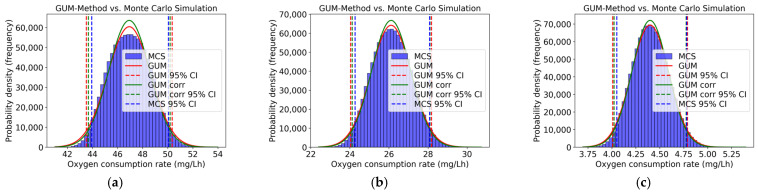
Distribution (GUM and MCS) of oxygen consumption rate (total respiration rate) (mg/Lh) for (**a**) 3,5-dichlorophenol at 0 mg/L, (**b**) 3,5-dichlorophenol at 10 mg/L, and (**c**) 3,5-dichlorophenol at 100 mg/L.

In order to validate the GUM results for the oxygen consumption rate (total respiration rate and rate due to heterotrophic respiration), a Monte Carlo Simulation was conducted. Figure 3 shows the distribution and the 95% coverage interval of the GUM method (with and without correlations) and the Monte Carlo Simulation in comparison. The calculations were performed for all concentration levels, but only three concentration levels are presented in Figure 3. Slight asymmetry can be observed at all concentration levels, and the coverage interval of the Monte Carlo Simulation and the GUM method, which consider the correlations, can be seen and are closer together.

The upper and lower coverage limits of the GUM method and the Monte Carlo Simulation, as well as the respective differences required for validation, are presented in Table 9. It can be seen that the numerical tolerance δ, both for with and without correlations, was met for all concentration levels, thus confirming the validation of the GUM method by the Monte Carlo Simulation. There is only one exception, the lower limit coverage interval 95% of 3,5-dichlorophenol 40 mg/L.

**Figure 4:**
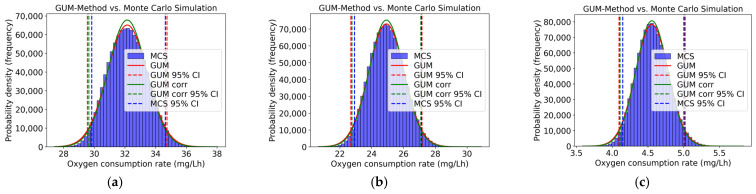
Distribution (GUM and MCS) of oxygen consumption rate (rate due to heterotrophic respiration) (mg/Lh) for (**a**) 3,5-dichlorophenol at 0 mg/L, (**b**) 3,5-dichlorophenol at 10 mg/L, and (**c**) 3,5-dichlorophenol at 100 mg/L.

Figure 4 illustrates the distribution and the 95% coverage interval of the GUM method (with and without correlations) and the Monte Carlo Simulation, comparing the two approaches. All concentration levels were calculated, but only three concentration levels are presented in Figure 4. Across all levels, a slight asymmetry can be observed. In most of these cases, the coverage interval of the Monte Carlo Simulation and the GUM method, which consider the correlations, are closer together.

Table 10 presents the upper and lower coverage limits of the GUM method and the Monte Carlo Simulation, as well as the respective difference required for validation. Also, here, it can be seen that the numerical tolerance δ was met for all concentration levels, thus confirming the validation of the GUM method via Monte Carlo Simulation. There is only one exception, the lower limit coverage interval 95% of 3,5-dichlorophenol 40 mg/L, the same as that for the total respiration rate. Within this concentration range, variability in the activated sludge and resulting asymmetry in the distribution of inhibition values become more pronounced. Since the GUM method relies on assumptions of linearity and normal distribution, these conditions reveal limitations in its applicability, which explains why validation against Monte Carlo Simulation failed in this case. Also, the lower limit coverage interval 95% of 3,5-dichlorophenol 100 mg/L for the uncorrelated GUM results cannot be validated. However, it is noteworthy that, in this case, considering correlations proved to be decisive. Only the correlated GUM results corresponded to the Monte Carlo Simulation. This shows that, under conditions of strong inhibition or high variability, correlations can significantly influence the validity of the uncertainty estimate, even though their effect is minor in most other cases. In such close cases, knowledge of the actual correlation values between the input variables is also important because generic assumptions do not necessarily lead to a reliable representation of the uncertainty.

#### 4.2.2. Percentage Inhibition

**Figure 5:**
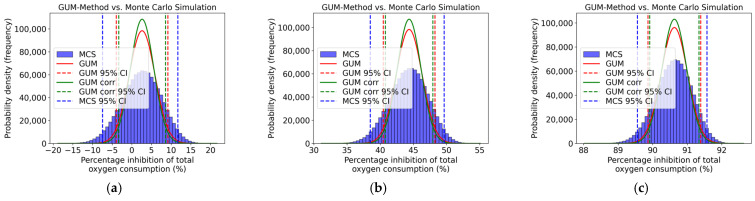
Distribution (GUM and MCS) of total oxygen consumption inhibition (%) for (**a**) 0.1 mg/L, (**b**) 10 mg/L, and (**c**) 100 mg/L of 3,5-dichlorophenol.

Also, for the percentage inhibition of total oxygen consumption, heterotrophic oxygen uptake, and oxygen uptake due to nitrification, the GUM results were validated using a Monte Carlo Simulation. Figure 5 compares the distribution and the 95% coverage interval obtained by using the GUM method and Monte Carlo Simulation. While the calculations were conducted for all concentration levels, only three concentration levels are displayed as examples in Figure 5. Also, here, a slight asymmetry can be observed in all concentration levels. Here, the coverage interval of the Monte Carlo Simulation and the uncorrelated GUM method are closer together. The fact that the coverage intervals of the Monte Carlo Simulation are closer to the uncorrelated GUM method results than to those of the correlated method indicates that including correlation terms in this case did not lead to a more realistic uncertainty estimate, but rather a distorted one. This is because the actual correlations between the input variables were unknown, so general recommendations from the JCGM were used instead. The result highlights that a GUM analysis incorporating correlation terms does not necessarily provide a more accurate approximation of the MCS benchmark. Its informative value depends largely on the availability of reliable correlation information. In the absence of this, the uncorrelated variant can provide a conservative yet methodologically robust estimate that is closer to the Monte Carlo Simulation results.

The upper and lower coverage limits of the GUM method and the Monte Carlo Simulation, as well as the respective difference required for validation, are presented in Table 11. It can be seen that the numerical tolerance, designated as δ, was not observed for any concentration level. The GUM method could not be validated by the Monte Carlo Simulation, thereby demonstrating that the GUM method underestimates the uncertainties.

**Figure 6:**
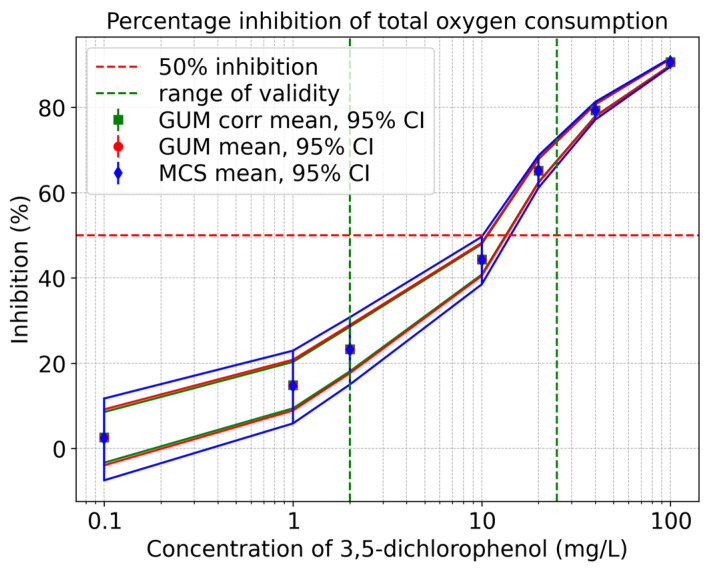
Qualitative comparison of the total oxygen consumption inhibition curves obtained using the GUM method and Monte Carlo Simulation within the validity range of 2–25 mg/L according to ISO 8192:2007 [1].

Figure 6 shows the inhibition curve for the percentage inhibition of total oxygen consumption. The solid red line signifies the uncorrelated GUM results (mean value and upper and lower 95% coverage interval), while the solid green line shows the correlated GUM results, and the sold blue line indicates the values derived from the Monte Carlo Simulation. It is evident from the figure that there is a slight asymmetry. Furthermore, it is apparent that, as the concentration of 3,5-dichlorophenol decreases, the GUM method tends to underestimate the associated uncertainties.

**Figure 7:**
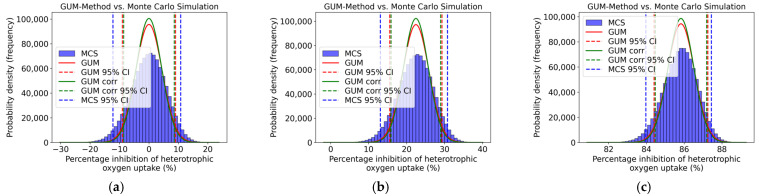
Distribution (GUM and MCS) of heterotrophic oxygen uptake inhibition (%) for (**a**) 0.1 mg/L, (**b**) 10 mg/L, and (**c**) 100 mg/L of 3,5-dichlorophenol.

Figure 7 shows the distribution and the 95% coverage interval of the GUM method and the Monte Carlo Simulation, comparing the two approaches. The calculations were performed for all concentration levels, but only three concentration levels are presented in Figure 7. An asymmetry can be observed for all concentration levels. Here, the coverage interval of the Monte Carlo Simulation and that of the uncorrelated GUM method are closer together.

The upper and lower coverage limits of the GUM method and the Monte Carlo Simulation, as well as the respective difference required for validation, are presented in Table 12. It can be seen that the numerical tolerance, designated as δ, was not observed for any concentration level, with the exception of a concentration of 100 mg/L 3,5 Dichlorophenol. The GUM method could not be validated by Monte Carlo Simulation, thereby demonstrating that the GUM method underestimates the uncertainties.

**Figure 8:**
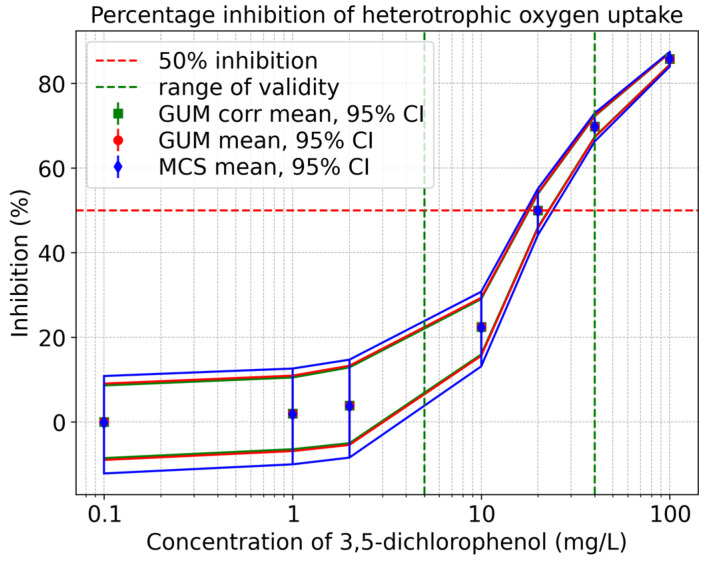
Qualitative comparison of the heterotrophic oxygen uptake inhibition curves obtained using the GUM method and Monte Carlo Simulation within the validity range of 5–40 mg/L according to ISO 8192:2007 [1].

Figure 8 displays inhibition curves for heterotrophic oxygen uptake. The solid red and solid green line represent the uncorrelated and correlated GUM results (mean value and upper and lower 95% coverage interval), respectively, and the solid blue line shows the results derived from the Monte Carlo Simulation. The plot shows a noticeable asymmetry. It is also apparent from this that, as the concentration of 3,5-dichlorophenol decreases, the GUM method tends to underestimate the associated uncertainties.

**Figure 9:**
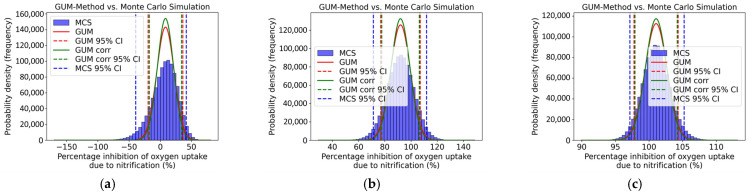
Distribution (GUM and MCS) of oxygen uptake due to nitrification inhibition (%) for (**a**) 0.1 mg/L, (**b**) 10 mg/L, and (**c**) 100 mg/L of 3,5-dichlorophenol.

Figure 9 compares the distribution and the 95% coverage interval obtained from the GUM method and the Monte Carlo Simulation. While the calculations were conducted for all concentration levels, only three concentration levels are displayed as examples in Figure 9. At all concentration levels, an asymmetry can be detected. Also, here, the coverage interval of the Monte Carlo Simulation and that of the uncorrelated GUM method are closer together.

The upper and lower coverage limits of the GUM method and the Monte Carlo Simulation, as well as the respective difference required for validation, are presented in Table 13. Also, here, it can be seen that the numerical tolerance, designated as δ, was not observed for any concentration level. The GUM method could not be validated by the Monte Carlo Simulation, thereby demonstrating that the GUM method underestimates the uncertainties.

**Figure 10:**
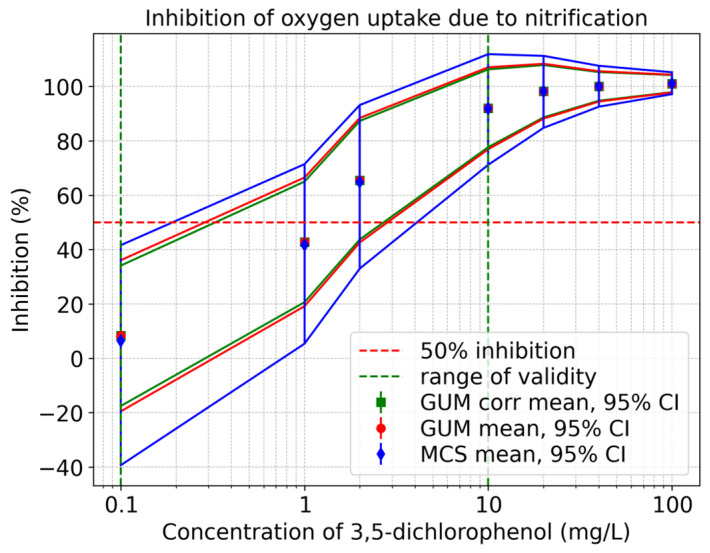
Qualitative comparison of the oxygen uptake due to nitrification inhibition curves obtained using the GUM method and Monte Carlo Simulation within the validity range of 0.1–10 mg/L according to ISO 8192:2007 [1].

Figure 10 shows inhibition curves for oxygen uptake due to nitrification. The solid red line signifies the uncorrelated GUM results (mean value and upper and lower 95% coverage interval), the solid green line represents the correlated GUM results, and the solid blue line indicates the values derived from the Monte Carlo Simulation. It is evident from the figure that there is an asymmetry. Furthermore, it is apparent that as the concentration of 3,5-dichlorophenol decreases, the GUM method tends to underestimate the associated uncertainties.

It is evident that the underestimation of measurement uncertainty is more pronounced at low concentrations. The biological variability of the activated sludge is a significant factor in this context. In practice, this means that effects within the detection limit range may not be recognized fully. Such underestimation carries the risk of minor yet ecologically significant changes appearing less relevant. In the context of ecotoxicological assessments, this can result in an inaccurate risk assessment due to the underestimation or overlooking of potentially harmful effects on aquatic systems. At low concentrations, minor interferences such as temperature fluctuations or the different microbial compositions of the activated sludge have a greater influence than the low inhibition effect, which leads to greater uncertainties. Consequently, there is a necessity for a greater number of repeat tests to be carried out, particularly at low concentrations.

The results confirm the statement from JCGM 100:2008 [13] that the GUM method provides accurate results when a linear measurement function and a normal distribution are present. However, JCGM 104:2009 [38] points out that the GUM method may be unsuitable if the measurement function is non-linear or if an asymmetric probability distribution exists. As mentioned in JCGM 104:2009 [38], it is sometimes difficult to determine in advance whether the conditions for applying the GUM method are met. The results for total respiration rate and heterotrophic respiration rate are almost symmetric, allowing the GUM results to be validated using the Monte Carlo Simulation. In contrast, the results for inhibitions (percentage inhibition of total oxygen consumption, heterotrophic oxygen uptake, and oxygen uptake due to nitrification) exhibit an asymmetric distribution, leading to an underestimation of uncertainties using the GUM method. Consequently, the GUM method can be applied to the total respiration rate and heterotrophic respiration rate, while a Monte Carlo Simulation is necessary for the inhibitions. The consideration of correlations in the GUM method also does not lead to significant improvements in the results.

## 5. Conclusions

1. The uncertainty budget shows that the most significant uncertainty contributors were identified to be the time interval of measurements ${∆}_{t}$, the tolerance temperature $\delta_{TT}$, and the accuracy of the oxygen concentration measurement $\delta_{\rho 1,G}$. For the oxygen consumption rate (total respiration rate/rate due to heterotrophic respiration), the tolerance temperature $\delta_{TT}$ contributed an average of approx. 46%/37%, the time interval ${∆}_{t}$ of the measurements contributed an average of approx. 36%/49%, and the accuracy of the oxygen concentration measurement $\delta_{\rho 1,G}$ had an average of approx. 10%/8%. These factors accounted for over 90% of the total uncertainty across all concentration levels. For the percentage inhibition (total oxygen consumption/heterotrophic oxygen uptake), the time interval ${∆}_{t}$ of measurements at a concentration of 3,5-dichlorophenol contributed an average of approx. 48%/51%, followed by the time interval ${∆}_{t}$ of blank control measurements, with approx. 22%/30%, as well as the accuracy of the oxygen concentration of blank control measurements $\delta_{\rho 1,G}$ (approx. 12%/8%) and the accuracy of the oxygen concentration measurements at a specific concentration of 3,5-dichlorophenol $\delta_{\rho 1,G}$, with approx. 12%/8%. These factors also accounted for over 90% of the total uncertainty across all concentration levels.
2. The GUM method was successfully validated for the oxygen consumption rate using the Monte Carlo Simulation. The absolute differences in the respective endpoints of the coverage intervals met the numerical tolerance δ, confirming its applicability for total and heterotrophic respiration rates.
3. However, the GUM method was not validated for the percentage inhibition because it underestimated uncertainties, particularly at lower concentrations. The results demonstrated an asymmetric probability distribution, making the Monte Carlo Simulation according to JCGM 101:2008 [21], a necessary alternative to the GUM method.
4. The influence of uncertainties varied depending on the concentration of 3,5-dichlorophenol. At low concentrations, biological variability and minor interferences have a disproportionate impact, leading to greater measurement uncertainty and highlighting the need for more repeat measurements in this range.
5. The findings support the statement from JCGM 100:2008 [13] that the GUM method provides reliable results under conditions of linearity and normal distribution. However, as noted in JCGM 104:2009 [38], the GUM method may be unsuitable for non-linear systems with asymmetric distributions. Consequently, while GUM is applicable for respiration rates, a Monte Carlo Simulation is required for accurate uncertainty assessment in inhibition studies.
6. The consideration of correlations in the GUM method does not lead to significant improvements in the results, but they can become critical under conditions of strong inhibition or high variability. In such situations, reliance on general correlation assumptions may distort the uncertainty estimate, whereas the Monte Carlo Simulation provides a more reliable reference.
7. The results indicate that, in practical application, particular attention should be paid to the precise recording of measurement time intervals, strict temperature control, regular calibration of oxygen probes, and repeat measurements at lower toxicant concentrations in order to reduce measurement uncertainties. Furthermore, the use of Monte Carlo Simulation in this case is recommended for a more accurate evaluation of measurement uncertainty. Based on our findings, we recommend adopting Monte Carlo Simulation as the preferred method, or at least as a standard complement to the GUM approach, for ISO 8192:2007-based testing, particularly at low and high toxicant concentrations. This would ensure more reliable uncertainty estimates and provide stronger support for environmental risk assessment and regulatory compliance.
8. Additionally, it is important to note that the present study applied the same assumptions to both the GUM and the Monte Carlo Simulation to enable a direct and meaningful comparison. Since the GUM framework is based on the assumptions of linearity, symmetry, and a normal distribution, these conditions were adopted for the MCS as well. While this ensured methodological consistency, it limited exploration of alternative distribution types. Future studies could address this limitation by applying generalized extreme value (GEV) or other non-normal distributions to MCS to more realistically capture uncertainty under extreme conditions (e.g., very low or high oxygen concentrations). This would provide further insights beyond the current scope and strengthen the methodological framework for uncertainty analysis in ecotoxicological testing.

## Figures and Tables

**Table 1:** Number of oxygen consumption rate measurements per concentration of 3,5-dichlorophenol for the determination of measurement uncertainty between the GUM method and Monte Carlo Simulation.

**Concentration** **3,5-Dichlorophenol (mg/L)**	Number of Oxygen Consumption Rate Measurements			
	$R_{T}$	$R_{TB}$	$R_{H}$	$R_{HB}$
0	-	92	-	92
0.1	46	-	46	-
1	46	-	46	-
2	38	-	38	-
10	46	-	48	-
20	36	-	38	-
40	34	-	34	-
100	27	-	28	-

**Table 2:** Relevant input quantities for the model equation, classification of the uncertainty type (GUM), determination of the probability distribution, and calculation of the standard uncertainty according to GUM.

Source of Uncertainty $X_{i}$	Unit	Type (GUM)	Distribution Type	Standard Uncertainty $u\left(x_{i}\right)$ (GUM)
$\rho_{1}$	mg/L	A	normal	$\frac{\rho_{1\_Std}}{\sqrt{n}}$
$\rho_{2}$	mg/L	A	normal	$\frac{\rho_{2\_Std}}{\sqrt{n}}$
${∆}_{T}$	°C	A	normal	$\frac{{∆}_{T\_Std}}{\sqrt{n}}$
${∆}_{t}$	min	A	normal	$\frac{{∆}_{t\_Std}}{\sqrt{n}}$
$\delta_{\rho 1,S},\delta_{\rho 2,S}$	mg/L	B	rectangular	$\frac{0.03}{\sqrt{3}}$
$\delta_{\rho 1,G}$	mg/L	B	rectangular	$\frac{0.015*\rho_{1\_mean}}{\sqrt{3}}$
$\delta_{\rho 2,G}$	mg/L	B	rectangular	$\frac{0.015*\rho_{2\_mean}}{\sqrt{3}}$
$\delta_{TS}$	°C	B	rectangular	$\frac{0.5}{\sqrt{3}}$
$\delta_{TT}$	°C	B	rectangular	$\frac{2}{\sqrt{3}}$
$\delta_{TG}$	°C	B	rectangular	$\frac{0.2}{\sqrt{3}}$

**Table 3:** Uncertainty budget: Analysis of uncertainty of the oxygen consumption rate (total respiration rate) based on ISO 8192:2007 [1] and Neunteufel et al.’s modification [2], as well as the application of the GUM methodology [13]. Example concentration of 3,5-dichlorophenol at 10 mg/L, with and without correlations.

Source of Uncertainty $X_{i}$	Unit	Distribution Type	Standard Uncertainty $u\left(x_{i}\right)$	Sensitivity Coefficient $c_{i}$	Contribution to Uncertainty (CTU) $u_{i}$	Percent of CTU Uncorr. (%)
$\delta_{TT}$	°C	rectangular	1.15470054	0.62005703	0.0715980	47.9
${∆}_{t}$	min	normal	0.25886013	−2.3567439	−0.610067	34.8
$\delta_{\rho 1,G}$	mg/L	rectangular	0.05953736	5.47322433	0.3258613	9.9
$\delta_{TS}$	°C	rectangular	0.28867513	0.62005703	0.7159801	3.0
$\delta_{\rho 2,G}$	mg/L	rectangular	0.01822042	−5.4732243	−0.0997245	0.9
$\delta_{\rho 1,S}$	mg/L	rectangular	0.01732051	5.47322433	0.0947990	0.8
$\delta_{\rho 2,S}$	mg/L	rectangular	0.01732051	−5.4732243	−0.094799	0.8
$\rho_{1}$	mg/L	normal	0.01620443	5.47322433	0.0886904	0.7
$\rho_{2}$	mg/L	normal	0.01328789	−5.4732243	−0.0727276	0.5
$\delta_{TG}$	°C	rectangular	0.11547005	0.62005703	0.1789950	0.5
${∆}_{T}$	°C	normal	0.04921672	0.62005703	0.0305171	0.1

**Table 4:** Uncertainty budget: Analysis of uncertainty of the oxygen consumption rate (rate due to heterotrophic respiration), based on ISO 8192:2007 [1] and Neunteufel et al.’s modification [2], as well as the application of the GUM methodology [13]. Example concentration of 3,5-dichlorophenol at 10 mg/L, with and without correlations.

Source of Uncertainty $X_{i}$	Unit	Distribution Type	Standard Uncertainty $u\left(x_{i}\right)$	Sensitivity Coefficient $c_{i}$	Contribution to Uncertainty (CTU) $u_{i}$	Percent of CTU Uncorr. (%)
${∆}_{t}$	min	normal	0.36439085	−2.1430424	−0.780905	49
$\delta_{TT}$	°C	rectangular	1.15470054	0.59169815	0.6832341	37.5
$\delta_{\rho 1,G}$	mg/L	rectangular	0.05946708	5.21546507	0.3101484	7.7
$\delta_{TS}$	°C	rectangular	0.28867513	0.59169815	0.1708085	2.3
$\rho_{1}$	mg/L	normal	0.01789532	5.21546507	0.0933323	0.7
$\delta_{\rho 1,S}$	mg/L	rectangular	0.01732051	5.21546507	0.0903345	0.7
$\delta_{\rho 2,S}$	mg/L	rectangular	0.01732051	−5.2154651	−0.0903345	0.7
$\delta_{\rho 2,G}$	mg/L	rectangular	0.01807467	−5.2154651	−0.0942678	0.7
$\delta_{TG}$	°C	rectangular	0.11547005	0.59169815	0.0683234	0.4
$\rho_{2}$	mg/L	normal	0.01104455	−5.2154651	−0.0576025	0.3
${∆}_{T}$	°C	normal	0.04550982	0.59169815	0.0269280	0.1

**Table 5:** Uncertainty budget: Analysis of uncertainty of the percentage inhibition of total oxygen consumption based on ISO 8192:2007 [1] and Neunteufel et al.’s modification [2], as well as the application of the GUM methodology [13]. Example concentration of 3,5-dichlorophenol at 10 mg/L, with and without correlations.

Source of Uncertainty $X_{i}$	Unit	Distribution Type	Standard Uncertainty $u\left(x_{i}\right)$	Sensitivity Coefficient $c_{i}$	Contribution to Uncertainty (CTU) $u_{i}$	Percent of CTU Uncorr. (%)
${∆}_{t}$	min	normal	0.25886013	5.02150919	1.2998685	44.7
${∆}_{t,\;FB}$	min	normal	0.09981651	−9.4438961	−0.9426567	23.5
$\delta_{\rho 1,G,\;FB}$	mg/L	rectangular	0.0584906	12.1233313	0.7091009	13.3
$\delta_{\rho 1,G}$	mg/L	rectangular	0.05953736	−11.661787	−0.6943121	12.7
Others	-	-	-	-	-	5.8

**Table 6:** Uncertainty budget: Analysis of uncertainty of the percentage inhibition of heterotrophic oxygen uptake, based on ISO 8192:2007 [1] and Neunteufel et al.’s modification [2], as well as the application of the GUM methodology [13]. Example concentration of 3,5-dichlorophenol at 10 mg/L, with and without correlations.

Source of Uncertainty $X_{i}$	Unit	Distribution Type	Standard Uncertainty $u\left(x_{i}\right)$	Sensitivity Coefficient $c_{i}$	Contribution to Uncertainty (CTU) $u_{i}$	Percent of CTU Uncorr. (%)
${∆}_{t,H}$	min	normal	0.36439085	6.66677184	2.4293106	49.9
${∆}_{t,\;FB,H}$	min	normal	0.21847779	−8.7413173	−1.9097836	30.9
$\delta_{\rho 1,G,\;FB,H}$	mg/L	rectangular	0.05924179	16.4709034	0.9757657	8.1
$\delta_{\rho 1,G,H}$	mg/L	rectangular	0.05946708	−16.224745	−0.9648382	7.9
Others	-	-	-	-	-	3.3

**Table 7:** Uncertainty budget: Analysis of uncertainty of the percentage inhibition of oxygen uptake, due to nitrification based on ISO 8192:2007 [1] and Neunteufel et al.’s modification [2], as well as the application of the GUM methodology [13]. Example concentration of 3,5-dichlorophenol at 100 mg/L, with and without correlations.

Source of Uncertainty $X_{i}$	Unit	Distribution Type	Standard Uncertainty $u\left(x_{i}\right)$	Sensitivity Coefficient $c_{i}$	Contribution to Uncertainty (CTU) $u_{i}$	Percent of CTU Uncorr. (%)
${∆}_{t,\;H}$	min	normal	2.40082317	−0.5004597	−1.2015152	53.7
${∆}_{t}$	min	normal	1.96813750	0.46906183	0.9231781	31.7
$\delta_{\rho 1,G,\;H}$	mg/L	rectangular	0.05792163	6.83298089	0.3957774	5.8
$\delta_{\rho 1,G}$	mg/L	rectangular	0.05801729	−6.6166093	−0.3838777	5.5
Others	-	-	-	-	-	3.2

**Table 8:** Uncertainty budget: Analysis of uncertainty of the percentage inhibition of oxygen uptake due to nitrification based on ISO 8192:2007 [1] and Neunteufel et al.’s modification [2], as well as the application of the GUM methodology [13]. Example concentration of 3,5-dichlorophenol at 0.1 mg/L with and without correlations.

Source of Uncertainty $X_{i}$	Unit	Distribution Type	Standard Uncertainty $u\left(x_{i}\right)$	Sensitivity Coefficient $c_{i}$	Contribution to Uncertainty (CTU) $u_{i}$	Percent of CTU Uncorr. (%)
${∆}_{t,\;H}$	min	normal	0.28893776	−24.347472	−7.034904	25.6
${∆}_{t}$	min	normal	0.12686679	50.7472072	6.4381354	21.5
${∆}_{t,FB}$	min	normal	0.09981651	−49.409595	−4.9318933	12.6
${∆}_{t,FB,H}$	min	normal	0.21847779	22.4733991	4.9099384	12.5
Others	-	-	-	-	-	8.9
$\delta_{\rho 1,G}$	mg/L	rectangular	0.05877865	−66.881531	−3.9312061	8
$\delta_{\rho 1,G,FB}$	mg/L	rectangular	0.0584906	63.4281531	3.7099509	7.1
$\delta_{\rho 1,G,\;H}$	mg/L	rectangular	0.05933027	45.8676785	2.7213417	3.8

**Table 9:** Validation of the GUM using MCS for uncertainty analysis of the oxygen consumption rate (total respiration rate) (mg/Lh).

Oxygen Consumption Rate (Total Respiration Rate) (mg/Lh)	GUM/GUM Corr Y−U(y) Y+U(y)	Monte Carlo Simulation $y_{low}$ $y_{high}$	GUM–MCS/ GUM Corr–MCS $d_{low}$ $d_{high}$	Validation of GUM/GUM Corr $d_{low}<\delta$ $d_{high}<\delta$
**Concentration 3,5-dichlorophenol: 0 mg/L**				
Lower limit coverage interval 95%	43.51/43.68	43.93	0.42/0.25	δ = 0.5 Validated
Upper limit coverage interval 95%	50.36/50.19	50.06	0.30/0.13	δ = 0.5 Validated
**Concentration 3,5-dichlorophenol: 0.1 mg/L**				
Lower limit coverage interval 95%	42.17/42.33	42.58	0.41/0.25	δ = 0.5 Validated
Upper limit coverage interval 95%	49.24/49.08	49.03	0.21/0.06	δ = 0.5 Validated
**Concentration 3,5-dichlorophenol: 1 mg/L**				
Lower limit coverage interval 95%	36.78/36.91	37.14	0.36/0.22	δ = 0.5 Validated
Upper limit coverage interval 95%	43.14/43.00	42.98	0.16/0.03	δ = 0.5 Validated
**Concentration 3,5-dichlorophenol: 2 mg/L**				
Lower limit coverage interval 95%	33.01/33.12	33.33	0.32/0.21	δ = 0.5 Validated
Upper limit coverage interval 95%	38.98/38.86	38.86	0.12/0.01	δ = 0.5 Validated
**Concentration 3,5-dichlorophenol: 10 mg/L**				
Lower limit coverage interval 95%	24.04/24.12	24.27	0.22/0.14	δ = 0.5 Validated
Upper limit coverage interval 95%	28.18/28.10	28.08	0.10/0.02	δ = 0.5 Validated
**Concentration 3,5-dichlorophenol: 20 mg/L**				
Lower limit coverage interval 95%	14.88/14.93	15.04	0.15/0.11	δ = 0.5 Validated
Upper limit coverage interval 95%	17.81/17.76	17.79	0.02/0.02	δ = 0.5 Validated
**Concentration 3,5-dichlorophenol: 40 mg/L**				
Lower limit coverage interval 95%	8.92/8.95	9.01	0.09/0.05	δ = 0.05 Not validated
Upper limit coverage interval 95%	10.48/10.45	10.44	0.03/0.00	δ = 0.05 Validated
**Concentration 3,5-dichlorophenol: 100 mg/L**				
Lower limit coverage interval 95%	4.01/4.02	4.05	0.04/0.03	δ = 0.05 Validated
Upper limit coverage interval 95%	4.79/4.78	4.78	0.01/0.01	δ = 0.05 Validated

**Table 10:** Validation of the GUM using MCS for uncertainty analysis of the oxygen consumption rate due to heterotrophic respiration) (mg/Lh).

Oxygen Consumption Rate (Rate Due to Heterotrophic Respiration) (mg/Lh)	GUM/GUM Corr Y−U(y) Y+U(y)	Monte Carlo Simulation $y_{low}$ $y_{high}$	GUM–MCS/ GUM Corr–MCS $d_{low}$ $d_{high}$	Validation of GUM/GUM Corr $d_{low}<\delta$ $d_{high}<\delta$
**Concentration 3,5-dichlorophenol: 0 mg/L**				
Lower limit coverage interval 95%	29.55/29.65	29.83	0.29/0.18	δ = 0.5 Validated
Upper limit coverage interval 95%	34.74/34.64	34.62	0.12/0.02	δ = 0.5 Validated
**Concentration 3,5-dichlorophenol: 0.1 mg/L**				
Lower limit coverage interval 95%	29.21/29.30	29.52	0.31/0.22	δ = 0.5 Validated
Upper limit coverage interval 95%	35.07/34.99	35.03	0.04/0.05	δ = 0.5 Validated
**Concentration 3,5-dichlorophenol: 1 mg/L**				
Lower limit coverage interval 95%	28.61/28.70	28.92	0.31/0.22	δ = 0.5 Validated
Upper limit coverage interval 95%	34.40/34.31	34.38	0.02/0.07	δ = 0.5 Validated
**Concentration 3,5-dichlorophenol: 2 mg/L**				
Lower limit coverage interval 95%	27.86/29.94	28.19	0.33/0.25	δ = 0.5 Validated
Upper limit coverage interval 95%	33.93/33.85	33.94	0.01/0.09	δ = 0.5 Validated
**Concentration 3,5-dichlorophenol: 10 mg/L**				
Lower limit coverage interval 95%	22.70/22.76	22.93	0.24/0.17	δ = 0.5 Validated
Upper limit coverage interval 95%	27.16/27.09	27.14	0.02/0.04	δ = 0.5 Validated
**Concentration 3,5-dichlorophenol: 20 mg/L**				
Lower limit coverage interval 95%	14.74/14.79	14.88	0.15/0.10	δ = 0.5 Validated
Upper limit coverage interval 95%	17.44/17.39	17.40	0.04/0.00	δ = 0.5 Validated
**Concentration 3,5-dichlorophenol: 40 mg/L**				
Lower limit coverage interval 95%	8.87/8.90	8.96	0.09/0.06	δ = 0.05 Not validated
Upper limit coverage interval 95%	10.54/10.51	10.51	0.02/0.01	δ = 0.05 Validated
**Concentration 3,5-dichlorophenol: 100 mg/L**				
Lower limit coverage interval 95%	4.10/4.11	4.15	0.05/0.04	δ = 0.05 Not v./Validated
Upper limit coverage interval 95%	5.01/5.00	5.02	0.01/0.02	δ = 0.05 Validated

**Table 11:** Validation of the GUM using MCS for uncertainty analysis of the percentage inhibition of total oxygen consumption (%).

Percentage Inhibition of Total Oxygen Consumption (%)	GUM/GUM Corr Y−U(y) Y+U(y)	Monte Carlo Simulation $y_{low}$ $y_{high}$	GUM–MCS/ GUM Corr–MCS $d_{low}$ $d_{high}$	Validation of GUM/GUM Corr $d_{low}<\delta$ $d_{high}<\delta$
**Concentration 3,5-dichlorophenol: 0.1 mg/L**				
Lower limit coverage interval 95%	−3.94/−3.33	−7.46	3.52/4.13	δ = 0.5 Not validated
Upper limit coverage interval 95%	9.17/8.56	11.73	2.56/3.17	δ = 0.5 Not validated
**Concentration 3,5-dichlorophenol: 1 mg/L**				
Lower limit coverage interval 95%	8.90/9.42	5.88	3.02/3.54	δ = 0.5 Not validated
Upper limit coverage interval 95%	20.82/20.30	22.94	2.12/2.64	δ = 0.5 Not validated
**Concentration 3,5-dichlorophenol: 2 mg/L**				
Lower limit coverage interval 95%	17.65/18.09	14.99	2.66/3.09	δ = 0.5 Not validated
Upper limit coverage interval 95%	28.97/28.54	30.76	1.79/2.22	δ = 0.5 Not validated
**Concentration 3,5-dichlorophenol: 10 mg/L**				
Lower limit coverage interval 95%	40.47/40.79	38.49	1.98/2.30	δ = 0.5 Not validated
Upper limit coverage interval 95%	48.25/47.93	49.65	1.39/1.71	δ = 0.5 Not validated
**Concentration 3,5-dichlorophenol: 20 mg/L**				
Lower limit coverage interval 95%	62.34/62.51	61.17	1.17/1.34	δ = 0.5 Not validated
Upper limit coverage interval 95%	68.01/67.84	68.71	0.71/0.88	δ = 0.5 Not validated
**Concentration 3,5-dichlorophenol: 40 mg/L**				
Lower limit coverage interval 95%	77.86/77.98	77.14	0.72/0.84	δ = 0.5 Not validated
Upper limit coverage interval 95%	80.80/80.69	81.30	0.50/0.62	δ = 0.5 Not validated
**Concentration 3,5-dichlorophenol: 100 mg/L**				
Lower limit coverage interval 95%	89.86/89.92	89.56	0.31/0.36	δ = 0.05 Not validated
Upper limit coverage interval 95%	91.39/91.34	91.57	0.18/0.24	δ = 0.05 Not validated

**Table 12:** Validation of the GUM using MCS for uncertainty analysis of the percentage inhibition of heterotrophic oxygen uptake (%).

Percentage Inhibition of Heterotrophic Oxygen Uptake (%)	GUM/GUM Corr. Y−U(y) Y+U(y)	Monte Carlo Simulation $y_{low}$ $y_{high}$	GUM–MCS/ GUM Corr–MCS $d_{low}$ $d_{high}$	Validation of GUM/GUM Corr $d_{low}<\delta$ $d_{high}<\delta$
**Concentration 3,5-dichlorophenol: 0.1 mg/L**				
Lower limit coverage interval 95%	−9.02/−8.58	−12.21	3.19/3.63	δ = 0.5 Not validated
Upper limit coverage interval 95%	9.03/8.59	10.82	1.79/2.22	δ = 0.5 Not validated
**Concentration 3,5-dichlorophenol: 1 mg/L**				
Lower limit coverage interval 95%	−6.92/−6.48	−10.06	3.13/3.57	δ = 0.5 Not validated
Upper limit coverage interval 95%	10.91/10.47	12.59	1.68/2.12	δ = 0.5 Not validated
**Concentration 3,5-dichlorophenol: 2 mg/L**				
Lower limit coverage interval 95%	−5.48/−5.06	−8.47	2.99/3.41	δ = 0.5 Not validated
Upper limit coverage interval 95%	13.26/12.85	14.68	1.42/1.84	δ = 0.5 Not validated
**Concentration 3,5-dichlorophenol: 10 mg/L**				
Lower limit coverage interval 95%	15.58/15.92	13.09	2.49/2.83	δ = 0.5 Not validated
Upper limit coverage interval 95%	29.33/28.99	30.75	1.42/1.76	δ = 0.5 Not validated
**Concentration 3,5-dichlorophenol: 20 mg/L**				
Lower limit coverage interval 95%	45.78/46.03	44.16	1.62/1.86	δ = 0.5 Not validated
Upper limit coverage interval 95%	54.12/53.88	55.11	0.99/1.23	δ = 0.5 Not validated
**Concentration 3,5-dichlorophenol: 40 mg/L**				
Lower limit coverage interval 95%	67.23/67.38	66.27	0.96/1.10	δ = 0.5 Not validated
Upper limit coverage interval 95%	72.38/72.24	72.97	0.59/0.73	δ = 0.5 Not validated
**Concentration 3,5-dichlorophenol: 100 mg/L**				
Lower limit coverage interval 95%	84.41/84.47	83.97	0.43/0.49	δ = 0.5 Validated
Upper limit coverage interval 95%	87.25/87.19	87.44	0.19/0.25	δ = 0.5 Validated

**Table 13:** Validation of the GUM using MCS for uncertainty analysis of the percentage inhibition of oxygen uptake due to nitrification (%).

Percentage Inhibition of Oxygen Uptake Due to Nitrification (%)	GUM/GUM Corr Y−U(y) Y+U(y)	Monte Carlo Simulation $y_{low}$ $y_{high}$	GUM–MCS/ GUM Corr–MCS $d_{low}$ $d_{high}$	Validation of GUM/GUM Corr $d_{low}<\delta$ $d_{high}<\delta$
**Concentration 3,5-dichlorophenol: 0.1 mg/L**				
Lower limit coverage interval 95%	−19.52/−17.55	−39.33	19.81/21.78	δ = 0.5 Not validated
Upper limit coverage interval 95%	36.08/34.11	41.62	5.53/7.51	δ = 0.5 Not validated
**Concentration 3,5-dichlorophenol: 1 mg/L**				
Lower limit coverage interval 95%	19.15/20.64	5.37	13.79/15.27	δ = 0.5 Not validated
Upper limit coverage interval 95%	66.50/65.02	71.43	4.92/6.41	δ = 0.5 Not validated
**Concentration 3,5-dichlorophenol: 2 mg/L**				
Lower limit coverage interval 95%	42.63/43.73	33.03	9.60/10.70	δ = 0.5 Not validated
Upper limit coverage interval 95%	88.43/87.33	93.19	4.76/5.86	δ = 0.5 Not validated
**Concentration 3,5-dichlorophenol: 10 mg/L**				
Lower limit coverage interval 95%	76.92/77.68	71.20	5.72/6.49	δ = 0.5 Not validated
Upper limit coverage interval 95%	107.07/106.30	111.91	4.84/5.60	δ = 0.5 Not validated
**Concentration 3,5-dichlorophenol: 20 mg/L**				
Lower limit coverage interval 95%	88.17/88.63	84.78	3.39/3.86	δ = 0.5 Not validated
Upper limit coverage interval 95%	108.35/107.88	111.27	2.92/3.38	δ = 0.5 Not validated
**Concentration 3,5-dichlorophenol: 40 mg/L**				
Lower limit coverage interval 95%	94.46/94.76	92.56	1.90/2.20	δ = 0.5 Not validated
Upper limit coverage interval 95%	105.61/105.31	107.62	2.01/2.31	δ = 0.5 Not validated
**Concentration 3,5-dichlorophenol: 100 mg/L**				
Lower limit coverage interval 95%	97.78/97.91	97.18	0.60/0.73	δ = 0.5 Not validated
Upper limit coverage interval 95%	104.33/104.20	105.23	0.90/1.03	δ = 0.5 Not validated

## Data Availability

The data presented in this study are available on request from the corresponding author.
